# A Muscle Fiber-Based Soft Hand Exoskeleton Control Strategy for Fine Manipulation: A Preliminary Investigation

**DOI:** 10.3390/biomimetics11090658

**Published:** 2026-09-12

**Authors:** Jia Yang, Chunyang Zhang, Ning Li, Jie Wen, Wenguang Yang, Wenyuan Chen

**Affiliations:** 1 School of Electrical Engineering and Automation, Tianjin University of Technology, Tianjin 300384, China; yangjia1992@email.tjut.edu.cn; 2 Tianjin Key Laboratory of New Energy Power Conversion, Transmission and Intelligent Control, Tianjin University of Technology, Tianjin 300384, China; 3 State Key Laboratory of Robotics and Intelligent Systems, Shenyang Institute of Automation, Chinese Academy of Sciences (CAS), Shenyang 110016, China; lining3@sia.cn; 4 Tianjin Key Laboratory for Advanced Mechatronic System Design and Intelligent Control, School of Mechanical Engineering, Tianjin University of Technology, Tianjin 300384, China; yangyang04150241@163.com; 5 National Demonstration Center for Experimental Mechanical and Electrical Engineering Education, Tianjin University of Technology, Tianjin 300384, China; 6 Sanying Motion Control Instruments Ltd., Tianjin 301700, China; jwen@symc-tec.com; 7 School of Electromechanical and Automotive Engineering, Yantai University, Yantai 264005, China; yangwenguang@ytu.edu.cn

**Keywords:** ultrasound-based perception, hand gesture recognition, finger motion control, machine learning, soft hand exoskeleton applications

## Abstract

While soft hand exoskeleton robots have approached human-level dexterity in terms of degrees of freedom, precise control methods for fine motor movements remain a significant challenge. Surface electromyography (sEMG) is widely employed in gesture recognition to enable patients to independently control a soft hand exoskeleton. However, individual finger control remains challenging through sEMG-based control due to the complexity of decoupling synergistic muscle activities. In this study, we propose a muscle fiber-based ultrasound perception strategy for fine hand motion recognition and soft hand exoskeleton control. Ultrasound imaging enables non-invasive visualization of forearm muscle morphology and provides information associated with underlying muscle-fiber activity. By reconstructing muscle morphology from ultrasound images, biologically relevant muscle-fiber features are extracted and fused to characterize fine hand movements. A lightweight Random Forest classifier is subsequently employed to map these biologically informed features to discrete hand actions, providing a computationally efficient recognition module for real-time control. To the best of our knowledge, publicly available ultrasound image datasets specifically designed for fine hand gesture recognition in rehabilitation applications remain limited. In the experiments, a dataset containing 21 hand gestures based on muscle ultrasound images was constructed to evaluate the proposed method. All data were collected from healthy participants as a preliminary proof-of-concept investigation. The results show that the proposed approach achieves an average recognition accuracy of 95.24% across three subjects in finger motion recognition. This preliminary study demonstrates the potential of machine learning-based ultrasound perception for improving fine hand gesture recognition and providing an intuitive control interface for soft hand exoskeletons, thereby enhancing their applicability in hand rehabilitation scenarios.

## 1. Introduction

Stroke is a leading cause of long-term motor disability worldwide. Although some stroke survivors regain function in their upper and lower limbs following treatment, the majority continue to suffer from hand dysfunction, and thus their activities of daily living are adversely affected [1,2,3]. Recently, exoskeleton robots specifically designed for hand rehabilitation have been extensively studied [4]. Hand exoskeletons can assist patients in performing repetitive hand-related tasks; therefore, neural plasticity of patients can be promoted through the reinforcement of functional motor pathways, ultimately facilitating the recovery of patients’ self-care abilities [5,6].

Soft exoskeletons have seen rapid development in recent years due to their inherent safety and adaptability. By integrating flexible structures and compliant actuators, soft hand exoskeletons facilitate safe physical interactions between patients and objects [7,8,9]. State-of-the-art designs feature high-degree-of-freedom systems capable of independently actuating the flexion and extension of individual fingers, as well as the abduction and adduction of the thumb [10]. These soft hand exoskeletons enable various grasping modalities essential for activities of daily living (ADLs), such as power grasps, precision pinches, and lateral pinches. Consequently, these devices achieve coordinated, dexterous movements that closely mimic human hand kinematics, significantly narrowing the functional gap between robotic assistants and the natural hand [11,12,13]. Unfortunately, the increase in system complexity and degrees of freedom (DoFs) significantly complicates user intent recognition, and thus control strategies in soft hand exoskeletons find it hard to fully harness the capabilities of such highly dexterous systems [14,15].

Most soft hand exoskeletons recognize the patient’s intent through physiological, voice and body movement signals. Physiological signals, including sEMG and Electroencephalography (EEG), have become increasingly prevalent in the development of hand exoskeletons owing to their ability to provide a seamless and intuitive human–robot interface. The sEMG-based control interfaces decode a patient’s hand movement intentions by capturing residual muscle activation signals via electrodes placed on the forearm. Although these interfaces have demonstrated success in gesture recognition for hand exoskeletons, the number of recognizable gestures remains severely constrained due to electrode displacement, skin impedance fluctuations, and muscular crosstalk [16,17,18,19]. EEG-based control interfaces are non-invasive brain–computer interfaces (BCIs) that directly translate cortical activity into actionable commands, proving highly valuable in hand exoskeleton control. However, as the inherently low signal-to-noise ratio of EEG fundamentally constrains the system’s performance, the practical application is typically restricted to binary movements, such as grasping and opening [20,21,22]. Voice-controlled exoskeleton systems utilize microphones to capture verbal commands, which are then processed by either onboard or cloud-based recognition modules to execute specific tasks. This approach is particularly beneficial for users with severe neuromuscular disorders who may struggle to generate sufficient or consistent muscle activation signals. However, the reliability of voice recognition remains highly sensitive to environmental noise, posing significant challenges for deployment in uncontrolled domestic or clinical settings [23,24,25]. Hand exoskeletons can also be controlled via sensors that monitor body movements, such as shoulder or foot motions. The necessity of mapping these unrelated body motions to hand gestures imposes a significant cognitive load and requires intensive pre-use training for the patient. Consequently, this control paradigm is typically restricted to a sparse command set and lacks the precision required for fine motor control and dexterous manipulation [26,27].

While the aforementioned methods have demonstrated feasibility in gesture-based control applications, they remain limited in recognizing diverse fine hand movements required for dexterous manipulation. Recently, muscle ultrasound has attracted increasing attention as a non-invasive sensing modality for hand motion recognition due to its capability of capturing muscle deformation patterns beneath the skin. Compared with surface-based physiological signals, ultrasound images provide spatial information related to muscle activation, which offers potential advantages for decoding fine finger movements. However, current decoding algorithms rely on features that lack biological relevance, which fail to adequately capture the intricate spatio-temporal dynamics of muscle deformation [28,29,30]. This means that ultrasound-based gesture recognition methods require a large amount of training images to obtain high-quality models. Unfortunately, publicly available ultrasound image datasets specifically designed for fine hand gesture recognition remain limited, particularly for rehabilitation-oriented human–machine interfaces. The lack of representative datasets restricts the development and evaluation of ultrasound-based control strategies for soft hand exoskeletons [31].

**Figure 1:**
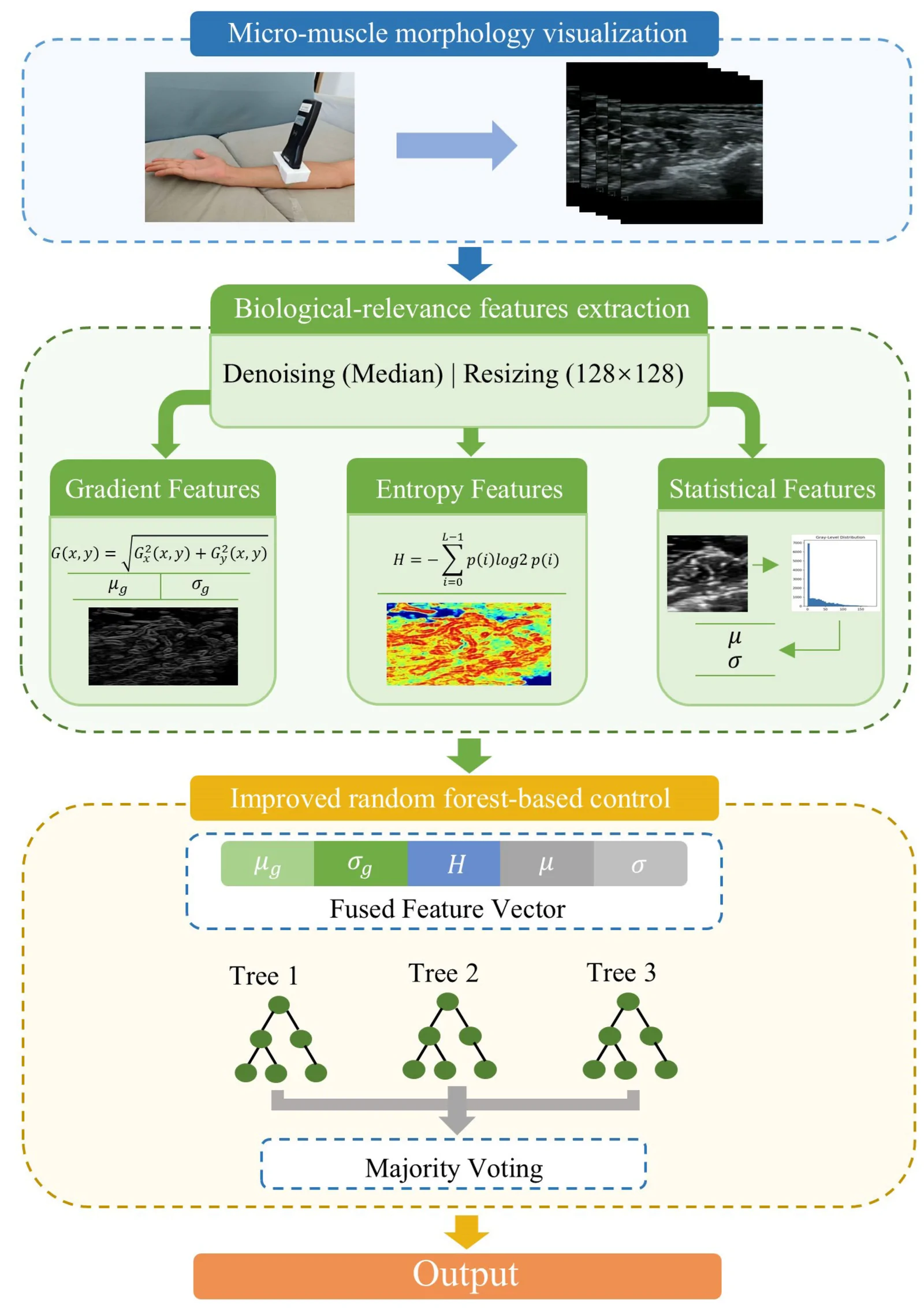
The muscle fiber dynamic-driven hand exoskeleton system. The system consists of three main components as follows: the micro-muscle morphology visualization, biological-relevance feature extraction and improved RF-based control.

In this study, an ultrasound-based hand motion intention recognition framework is developed for soft hand exoskeleton control, as shown in Figure 1. In contrast to conventional control strategies that primarily rely on surface physiological signals, ultrasound sensing is employed to capture forearm muscle deformation information for fine hand gesture recognition. As a preliminary investigation in healthy participants, this study aims to investigate the feasibility of ultrasound-based perception in rehabilitation-oriented human–machine interfaces. A dedicated ultrasound image dataset comprising 21 hand gestures is established. Furthermore, a machine learning-based recognition strategy incorporating biologically relevant multi-feature fusion and a lightweight Random Forest classifier is developed to decode ultrasound image features into hand gesture intentions.

## 2. Materials and Methods

### 2.1. Micro-Muscle Morphological Sensing

**Figure 2:**
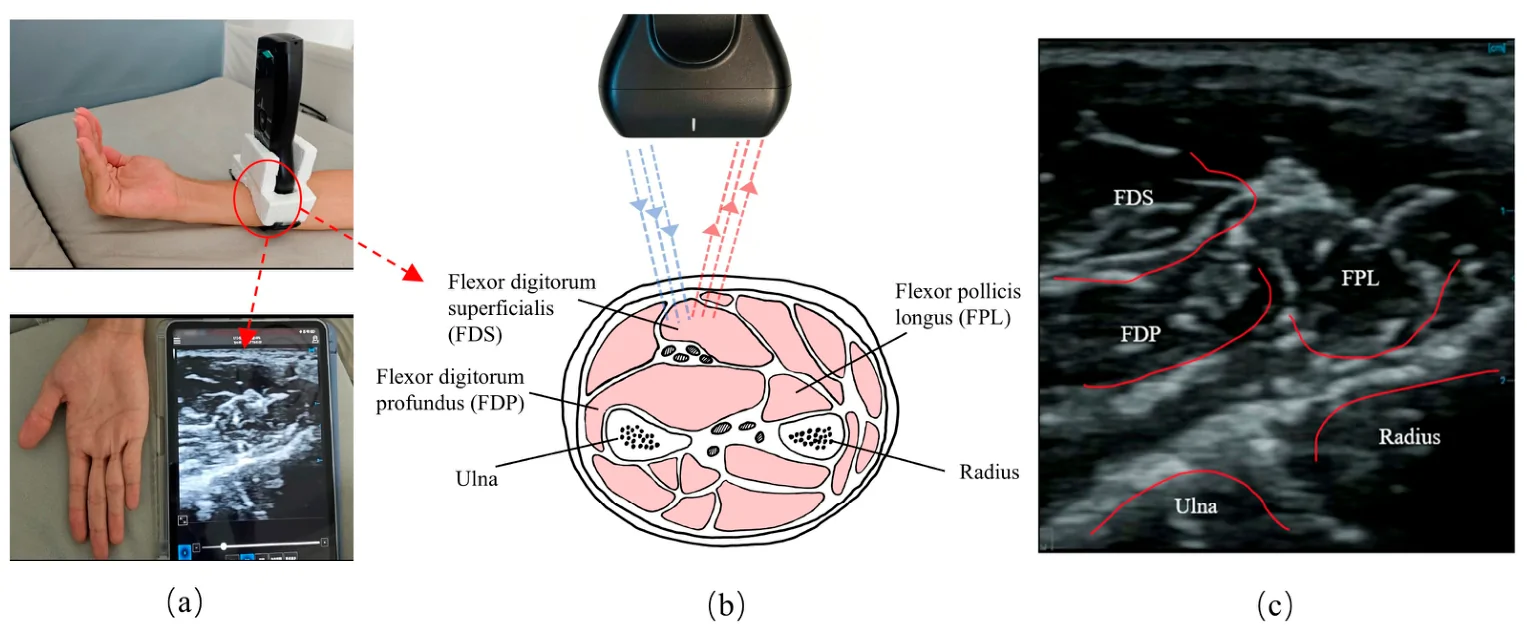
The proposed ultrasound-based micro-muscle morphological sensing system. (**a**) The sensing system contains an ultrasound probe attached to the wrist and a data processing unit. (**b**) The ultrasound probe can transmit high-frequency acoustic waves into biological tissues and receive the echoes reflected from tissue boundaries. The key muscle groups involved in hand movements are shown in the cross-sectional anatomical diagram of the human forearm, including the flexor digitorum superficialis (FDS), flexor digitorum profundus (FDP), and flexor pollicis longus (FPL), as well as the radius and ulna. (**c**) An example of the ultrasound image with annotated hand muscles.

The execution of hand movements is under the coordinated control of the forearm and wrist muscle groups. During hand movements, the corresponding muscles contract or relax, leading to changes in muscle thickness, shape, and internal structure. These microscopic changes are difficult to capture using traditional sensing methods, such as sEMG [32,33]. Therefore, we propose an ultrasound-based micro-muscle morphological sensing system as shown in Figure 2a. The system contains an ultrasound probe attached to the wrist and a data processing unit. The probe can transmit high-frequency acoustic waves into biological tissues and receive the echoes reflected from tissue boundaries, as shown in Figure 2b. Because different tissues exhibit different acoustic impedances, the reflected signals vary in intensity, and thus the microscopic morphology of muscles can be detected. The data processing unit integrates these microscopic muscle data into grayscale images, as shown in Figure 2c. Fine hand movements are generated through the coordinated activation of multiple forearm muscles. Among them, the flexor digitorum superficialis (FDS) primarily flexes the proximal interphalangeal joints of the fingers, the flexor digitorum profundus (FDP) mainly contributes to flexion of the distal interphalangeal joints, and the flexor pollicis longus (FPL) is responsible for thumb flexion. Different hand gestures involve different activation patterns and coordination of these muscles. Obviously, the contraction of forearm muscles leads to changes in the spatial arrangement of muscle fibers and surrounding tissues. Consequently, the ultrasound images of the forearm exhibit observable variations in texture patterns, grayscale distribution, and structural features.

To ensure consistent ultrasound imaging conditions, the ultrasound probe was fixed to the participant’s forearm using a custom-made fixation holder constructed from rigid foam and elastic straps, which effectively minimized probe displacement and rotation during data acquisition. A force sensor integrated into the fixation system continuously monitored the probe-skin contact force, allowing the contact pressure to be adjusted before recording and maintained within a stable range throughout the experiment. Furthermore, each recording session consisted of continuous acquisition of multiple predefined hand movements without repositioning the probe, thereby maintaining consistent probe orientation and contact conditions across all collected ultrasound images.

The proposed ultrasound-based sensing system provides an effective approach for monitoring muscle activity during hand movements. The ultrasound image variations contain discriminative information related to different hand movements. Then, various feature extraction techniques can be employed to quantify these variations, such as texture descriptors derived from the Gray-Level Co-occurrence Matrix and other statistical image features. The extracted features can be used as input to machine learning classifiers for hand movement recognition. However, traditional recognition methods lack biological interpretability, which cannot meet the fine motor control requirements of soft hand exoskeletons in terms of training efficiency and identifiable hand movements. To recognize the fine hand movement, this study proposes biological-relevance muscle feature extraction and a fine hand movement recognition model, which are described in Section 2.2 and Section 2.3, respectively.

### 2.2. Biological-Relevance Muscle Feature Extraction

Ultrasound images collected from the forearm muscles are subsequently filtered using a 3×3 median filter to suppress speckle noise while preserving local structural information. The filtering process can be expressed as:

(1) $$I\left(x,y\right)=median\{I_{raw}(x+i,y+j)|i,j\in \{-1,0,1\}\}$$

where $I_{raw}$ and I denote the intensity values of the raw and filtered ultrasound images, respectively. Subsequently, all images are resized to a standardized resolution of 128×128 pixels using bilinear interpolation to ensure consistent input dimensions for feature extraction. This resolution can effectively reduce computational cost while retaining sufficient muscle texture and deformation information for subsequent motion recognition.

To effectively characterize muscle deformation patterns in ultrasound images, multiple complementary features are extracted from each ultrasound image. Because ultrasound muscle images exhibit notable variations in muscle texture, intensity distribution, and structural shape during different hand gesture execution processes, three types of hand-crafted features are employed in this study, including gradient features, entropy-based texture features, and statistical intensity features. These features are designed to capture both local intensity variations and global structural information, providing a comprehensive representation of ultrasound muscle dynamics.

#### 2.2.1. Gradient Features

The gradient feature is widely used in the field of traditional image processing, where it can describe structural variations and edge information in images. In this study, muscle contraction during hand movements leads to deformation of muscle fibers and boundaries, resulting in significant changes in the grayscale gradient in ultrasound images. Therefore, the gradient feature can effectively capture the morphological characteristics of hand muscles.

In this study, the horizontal gradients $G_{x}$ and vertical gradients $G_{y}$ of the image can be calculated as follows:

(2) $$G_{x}(x,y)=I\left(x+1,y\right)-I\left(x-1,y\right)$$

(3) $$G_{y}(x,y)=I(x,y+1)-I\left(x,y-1\right)$$

Then, the gradient magnitude at each pixel G can be defined as follows:

(4) $$G(x,y)=\sqrt{G_{x}^{2}\left(x,y\right)+G_{y}^{2}(x,y)}$$

To describe the overall gradient distribution of the image, the mean $\mu_{g}$ and standard deviation $\sigma_{g}$ of the gradient magnitude are extracted as gradient descriptors.

(5) $$\mu_{g}=\frac{1}{N}\sum_{i=1}^{N}G_{i}$$

(6) $$\sigma_{g}=\sqrt{\frac{1}{N}\sum_{i=1}^{N}{{(G}_{i}-\mu_{g})}^{2}}$$

where N denotes the total number of pixels and $G_{i}$ denotes the gradient magnitude at the i-th pixel.

#### 2.2.2. Entropy

Entropy is commonly used to quantify the randomness or complexity of grayscale distributions in an image. In this study, the texture of specific muscles in ultrasound images exhibits complex changes during muscle contraction. Therefore, this study extracts the entropy features of ultrasound images to dynamically track texture changes in hand muscles. The entropy value is extracted as a texture feature for gesture classification, and it is defined as follows:

(7) $$H=-\sum_{i=0}^{L-1}p(i)\log_{2}p(i)$$

where L denotes the number of grayscale levels, and $p\left(i\right)$ denotes the probability of the grayscale level i in the image histogram, which can be calculated through the following equation:

(8) $$p\left(i\right)=\frac{n_{i}}{N}$$

where $n_{i}$ denotes the number of pixels in the image with a grayscale level equal to i and N denotes the total number of pixels.

#### 2.2.3. Statistical Features

Statistical features are widely used to describe the global intensity distribution of the image. In this study, muscle contraction alters the reflectivity of surrounding tissues, resulting in changes in the overall brightness and contrast of the ultrasound image. Therefore, statistical descriptors indirectly describe the microscopic morphological changes during muscle contraction.

In this study, the statistical features include the mean intensity and the standard deviation of the ultrasound images. The mean intensity of the image μ is defined as follows:

(9) $$\mu =\frac{1}{N}\sum_{i=1}^{N}I_{i}$$

and the standard deviation of the image σ is defined as follows:

(10) $$\sigma =\sqrt{\frac{1}{N}\sum_{i=1}^{N}{\left(I_{i}-\mu \right)}^{2}}$$

where $I_{i}$ represents the grayscale intensity of the ultrasound image in the i-th pixel and N is the total number of pixels in the image. The mean intensity reflects the overall brightness level, while the standard deviation describes the contrast variation in the ultrasound image.

### 2.3. Fine Hand Movement Recognition

In this study, an RF classifier is employed for fine hand movement recognition. RF is an ensemble learning method that constructs multiple decision trees during training and determines the final prediction by majority voting. Compared with a single decision tree, RF generally provides better robustness and stronger generalization performance. Rather than modifying the RF algorithm itself, the proposed method improves the input representation by integrating multiple biologically relevant muscle features. The gradient features, entropy feature, and statistical features are concatenated to form a unified feature vector F. The fused feature vector utilizes complementary information from different feature types to provide a more comprehensive description of muscle deformation during contraction. The resulting feature vector is used as the input to the RF classifier. The classifier is implemented using the Scikit-learn library with 200 decision trees, and the random seed is fixed at 42 to ensure reproducibility. The training and validation sets are merged to train the final model, while the independent testing set is reserved exclusively for performance evaluation. Unless otherwise specified, all remaining parameters are kept at their default settings in Scikit-learn. The feature fusion process is formulated as follows:

(11) $$F=[\mu_{g},\sigma_{g},H,\mu ,\sigma ]$$

During the training process, the classifier learns the mapping between the extracted feature vectors and corresponding gesture labels. The trained model is then used to predict the gesture category of unseen ultrasound images. By combining multi-feature representation with the RF classifier, the proposed framework achieves reliable gesture recognition based on ultrasound muscle images. The gesture recognition performance of the proposed method was evaluated using the testing dataset. The overall classification accuracy is defined as

(12) $$Accuracy=\frac{N_{correct}}{N_{total}}\times 100\%$$

where $N_{correct}$ is the number of correctly predicted samples and $N_{total}$ denotes the total number of samples in the testing dataset. Three subjects took part in our experiments. Each subject has 2100 standardized ultrasound frames covering all 21 gesture categories. The classifier reaches 98.10% accuracy on Subject 1. It records 95.71% accuracy on Subject 2, and 91.92% accuracy on Subject 3. The average accuracy across all three participants stands at 95.24%. These results demonstrate that the proposed feature extraction and fusion strategy can effectively capture the discriminative characteristics of muscle deformation patterns in ultrasound images, enabling reliable hand gesture classification.

### 2.4. Soft Hand Exoskeleton Control

**Figure 3:**
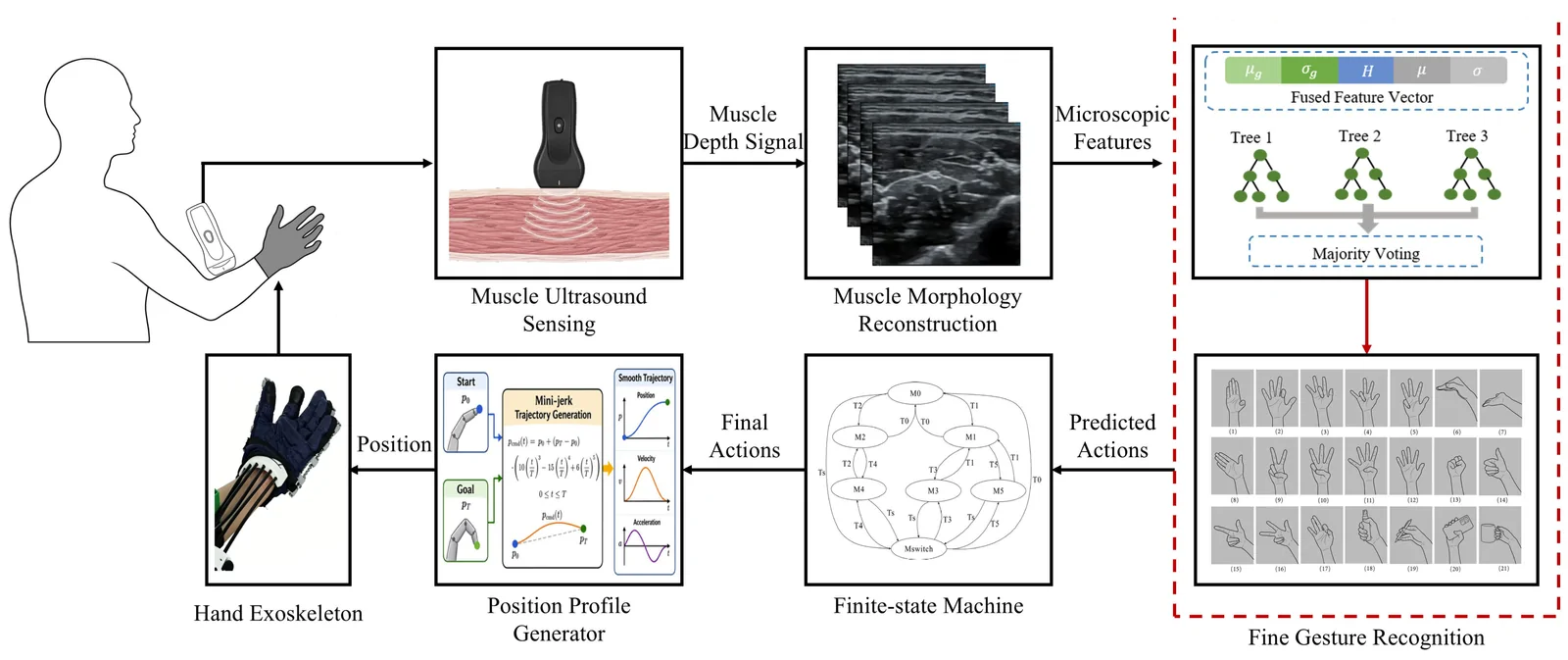
The control framework of the ultrasound-based soft hand exoskeleton system.

**Figure 4:**
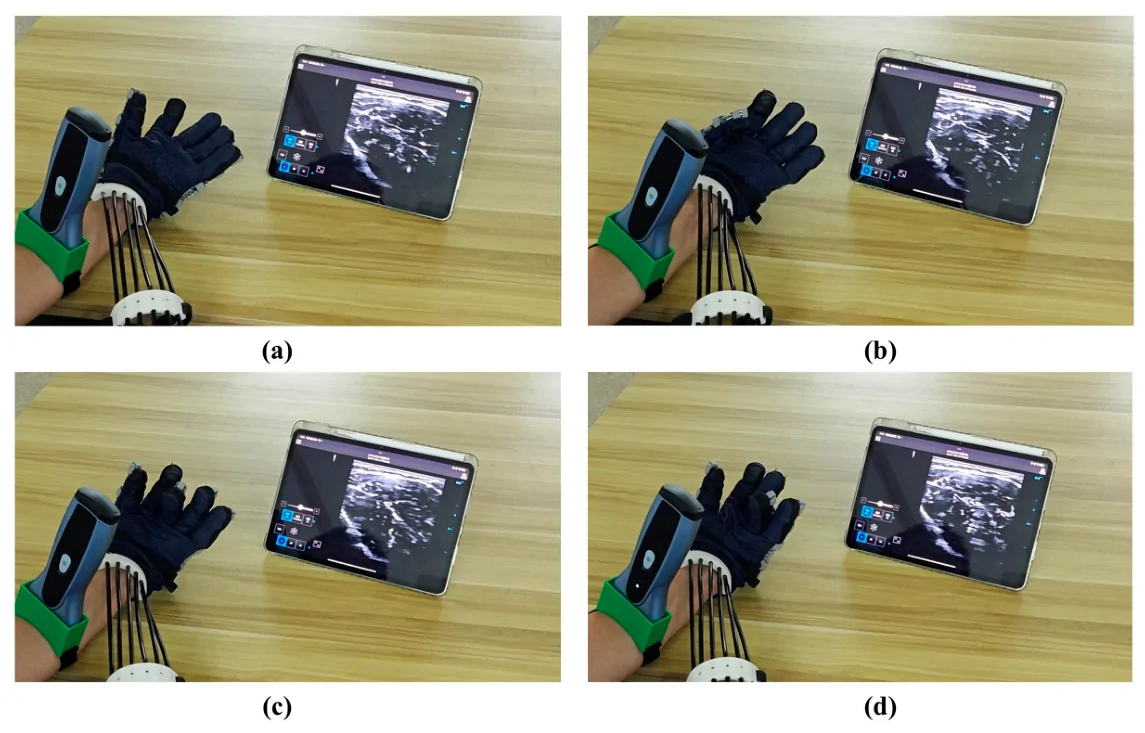
Illustration of the four target gestures for soft hand exoskeleton control. (**a**) Abduction of the fingers, (**b**) thumb flexed while extending others, (**c**) middle finger flexion, (**d**) ring finger flexion.

In this study, the soft hand exoskeleton reported in our previous study [26,34] is used to test the proposed method. Figure 3 illustrates the overall control framework for fine hand manipulation. The muscle ultrasound sensing module first acquires muscle depth signals during voluntary hand movements, from which muscle morphology is reconstructed and microscopic muscle-fiber features are extracted. These features are fused and fed into a fine gesture recognition module based on an ensemble of decision trees, yielding the predicted hand action. The recognized action is subsequently mapped to the corresponding finger motion through the finite-state machine (FSM), while the position profile generator converts the desired motion into actuator trajectories for the hand exoskeleton. During execution, the exoskeleton provides the user with the corresponding assisted hand motion, thereby establishing an integrated pathway from muscle-fiber sensing and intention recognition to exoskeleton motion execution, as shown in Figure 4 and Supplementary Video.

While high recognition accuracy is achieved by the proposed method, instantaneous misclassifications or noise in ultrasound imaging may cause the exoskeleton to execute abrupt and unintended actions. Therefore, the soft hand exoskeleton control is controlled by the finite-state machine to ensure the safety and stability of the soft hand exoskeleton during continuous manipulation. The transition of exoskeleton states relies not only on the current gesture recognition result but also on the previous state. The state transition logic is defined based on the continuity of human hand movements. Let $S_{t}\in \{G_{1},G_{2},\ldots ,G_{n}\}$ denote the current intended gesture at time t, and $S_{t-1}$ denote the state executed by the exoskeleton at time t−1. The exoskeleton only executes the action corresponding to $S_{t}$ if the recognition result satisfies one of the following conditions:

(1) Direct Transition: The recognized gesture remains consistent for a predefined number of consecutive frames, which filters out transient noise.

(2) Logical Sequence Transition: The recognized gesture $S_{t}$ represents a logically feasible subsequent motion from the current state $S_{t-1}$, avoiding physically impossible transitions.

If a sudden, isolated gesture is detected that deviates from the continuous sequence or fails to meet the threshold, the exoskeleton maintains the previous state $S_{t-1}$ until a stable and valid gesture is confirmed. The FSM improves the system’s robustness to transient fluctuations and prevents the exoskeleton from making erroneous movements that could accidentally injure the user.

Once the current hand action $S_{t}$ is determined by the FSM, the soft hand exoskeleton performs the corresponding movement. A position profile generator is used to construct the commanded motor position $p_{cmd}(t)$ based on the minimum-jerk principle, thereby generating a smooth motor trajectory. The commanded motor position is expressed as

(13) $$p_{cmd}\left(t\right)=p_{0}+\left(p_{T}-p_{0}\right)\left(10{\left(\frac{t}{T}\right)}^{3}-15{\left(\frac{t}{T}\right)}^{4}+6{\left(\frac{t}{T}\right)}^{5}\right)$$

where $p_{0}$ and $p_{T}$ denote the initial and target motor positions at t=0 and t=T, respectively, and T denotes the duration of the action period.

During the action period, the commanded motor trajectory is tracked using a proportional-derivative (PD) position controller. The control law is given by

(14) $$v_{cmd}=k_{p}\left(p_{cmd}-p\right)-k_{d}v$$

where $k_{p}$ and $k_{d}$ are the proportional gain and derivative gain; $p_{cmd}\;$and p denote the commanded and measured motor positions, respectively; and $v_{cmd}$ and v denote the commanded and measured motor velocities, respectively. The generated motor trajectory is subsequently converted into actuator commands to drive the soft hand exoskeleton to perform the corresponding hand movement.

## 3. Results

### 3.1. Experimental Setup and Data Acquisition

The study was conducted in accordance with the Declaration of Helsinki, and approved by the Ethics Committee of Science and Technology Ethics Committee of Shenyang Institute of Automation, Chinese Academy of Sciences (protocol code SIA-KJLLSC-2023-001 and date of approval 23 October 2023). The ultrasound data were collected using a portable ultrasound imaging device equipped with a linear L40 probe with a frequency range of 5–12 MHz. During the acquisition process, the imaging depth was set to 3 cm and the operating frequency was adjusted to 10.5 MHz. The ultrasound images were streamed and recorded on a personal computer at a frame rate of 30 Hz. To ensure stable image acquisition, the ultrasound probe was gently but firmly fixed to the subject’s forearm using a custom support bracket and elastic straps. The transducer was positioned perpendicular to the skin surface near the distal radius of the forearm, behind the pronator muscle region. This location was selected to ensure that the muscle thickness remained within the effective detection range of the ultrasound probe while still allowing observable ultrasound image variations caused by muscle contraction.

**Figure 5:**
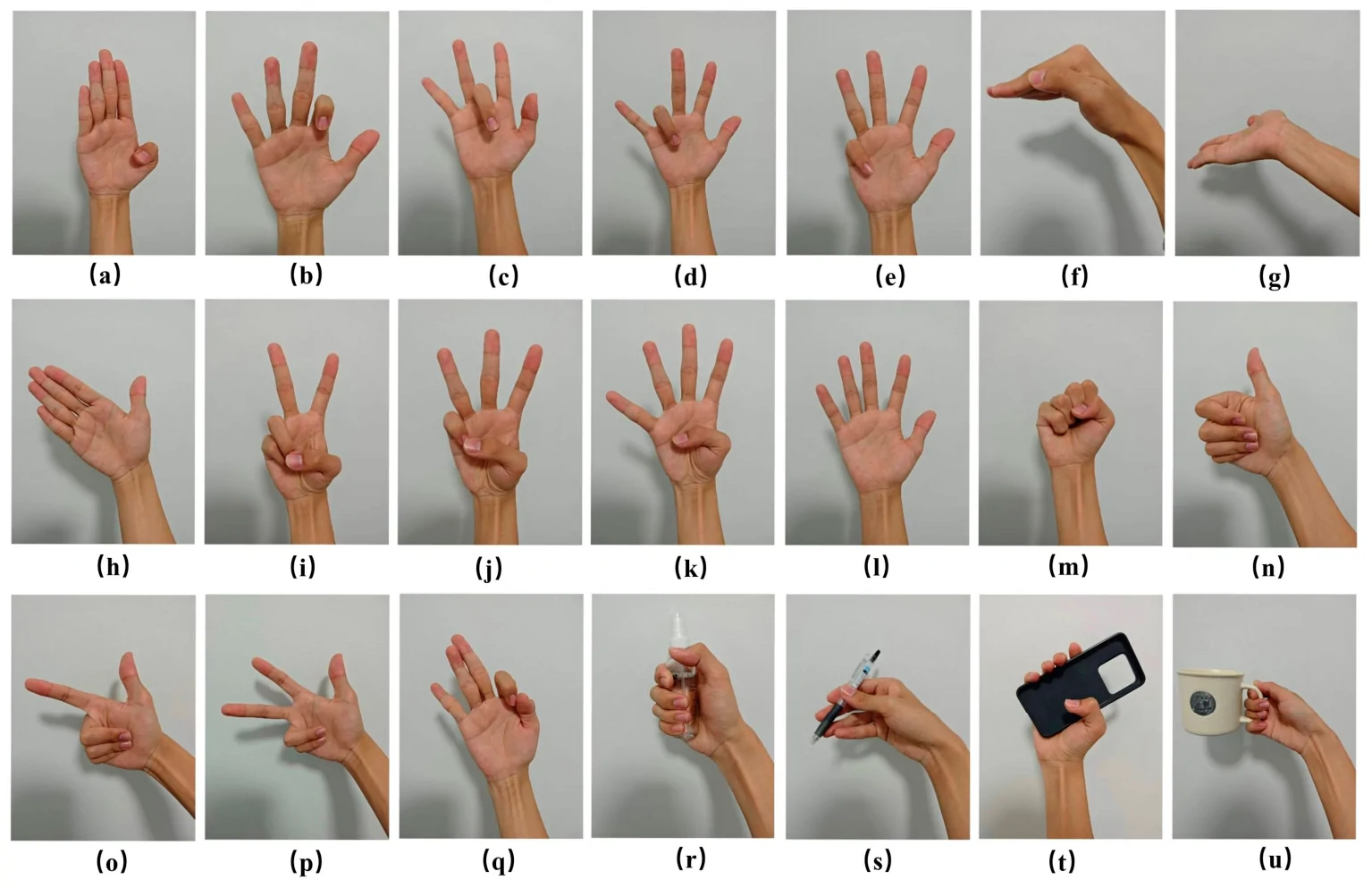
The 21 movements included in the experiment. (**a**) Thumb flexion; (**b**) index finger flexion; (**c**) middle finger flexion; (**d**) ring finger flexion; (**e**) little finger flexion; (**f**–**h**) wrist flexion, extension and ulnar deviation; (**i**) index and middle finger extension; (**j**) index, middle and ring finger extension; (**k**) thumb flexed while extending other fingers; (**l**) abduction of the fingers; (**m**) fingers flexed together in a fist; (**n**) thumb extended while flexing other fingers; (**o**) thumb and index finger extended while flexing other fingers; (**p**) thumb, index and middle finger extended while flexing other fingers; (**q**) middle, ring and little finger extended while flexing other fingers; (**r**) grasping a cylinder; (**s**) pinching a pen; (**t**) grasping a rectangle; (**u**) grasping a handle.

During the experiment, three subjects were recruited. All subjects were right-handed, with no history of neuromuscular or skeletal disorders and normal upper-limb function. The detailed information is summarized in Table 1. The subject sat comfortably on a chair with the forearm placed in a natural resting position. The probe orientation was aligned perpendicular to the muscle fibers of the flexor carpi radialis. The subject performed predefined hand gestures according to visual cues displayed on a computer screen, while ultrasound images were continuously recorded. A total of 21 hand gestures were designed in this study. These gestures included both basic hand movements and several functional grasping actions commonly used in daily activities, such as grasping a pen or holding a cup. Each gesture was maintained for 5 s, followed by a 3 s rest period before the next gesture began. One complete sequence contained all 21 gestures. After each sequence, the subject rested for approximately 5–10 min to avoid muscle fatigue. The entire acquisition process was repeated ten times. The specific 21 hand gestures are shown in Figure 5.

Considering the subject’s reaction time, a temporal deviation may occur between the visual cue and the actual execution of the gesture. Typically, the subject required approximately 0.5–1 s to reach a stable gesture state, and in some cases the gesture might be released slightly before the recording ended. To ensure that only stable muscle contraction periods were used for analysis, the first and the last seconds of each gesture segment were discarded. Therefore, only the data between the 2nd and 4th seconds were retained.

The dataset was partitioned at the trial level to avoid potential data leakage. Specifically, each gesture was performed in 10 independent trials, and 10 ultrasound images were uniformly sampled from the stable contraction period of each trial, resulting in 100 images per gesture and 2100 images across 21 gesture classes. Although the sampled images were acquired at different time points, images within the same trial shared similar acquisition conditions, including probe positioning, probe–skin contact, and muscle contraction state, and therefore exhibited substantial intra-trial correlation. Randomly splitting individual images could consequently place highly correlated samples from the same trial into different subsets, leading to an overly optimistic estimation of recognition performance. Therefore, all images from the same trial were kept within a single subset, with eight trials used for training, one for validation, and one for testing, resulting in an 8:1:1 split. This ratio was determined directly by the experimental design of 10 independent trials per gesture rather than selected to optimize recognition performance, while the trial-level separation ensured that the testing data originated from a previously unseen trial.

### 3.2. Feature Visualization and Interpretation

**Figure 6:**
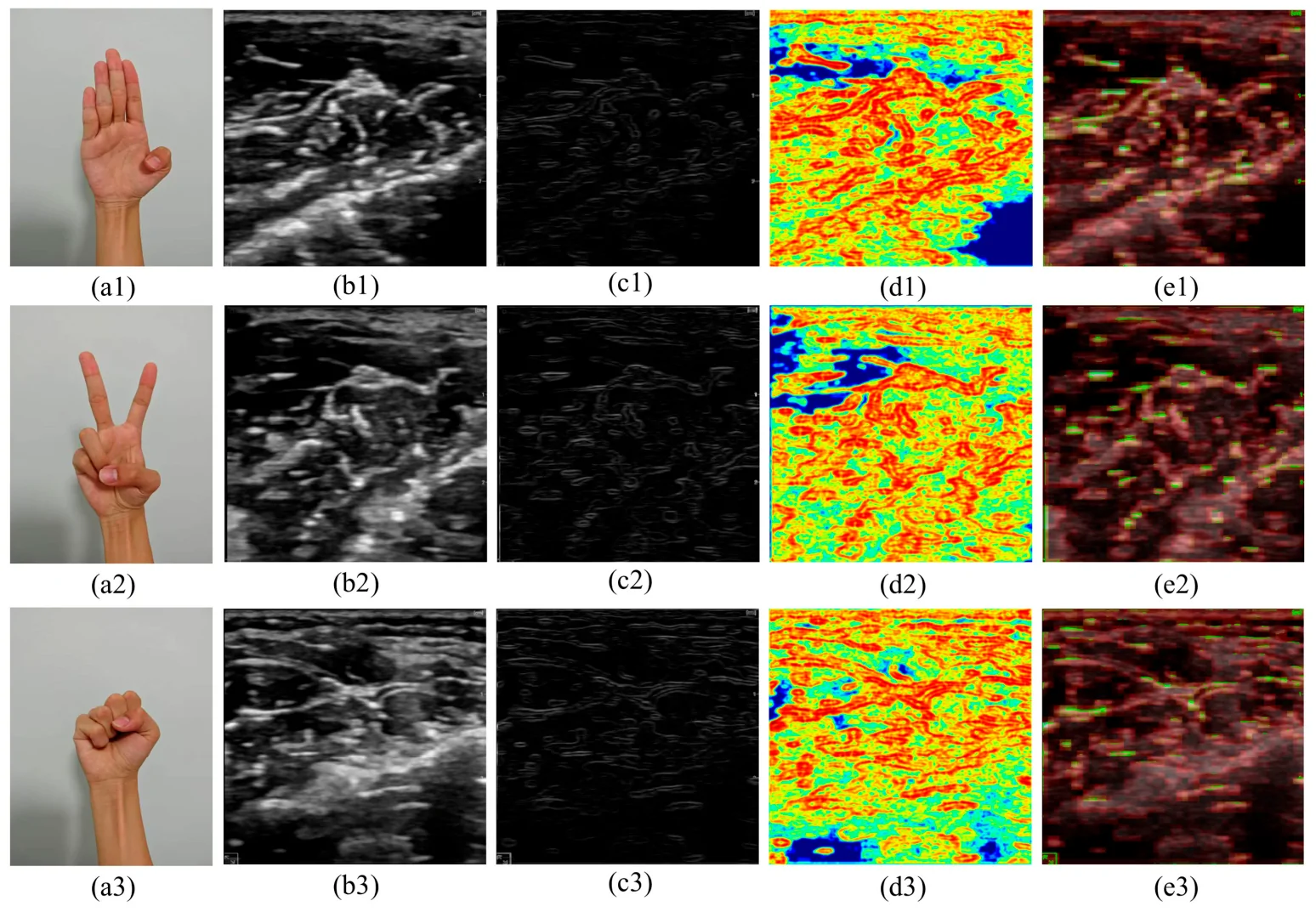
Visualization results of feature extraction for Gesture 01, 09, and 13 from Subject 1. (**a**) Gesture images; (**b**) Raw ultrasound frames of the gesture; (**c**) Gradient maps of the gesture; (**d**) Entropy heatmaps of gesture; (**e**) Feature fusion maps of the gesture.

**Figure 7:**
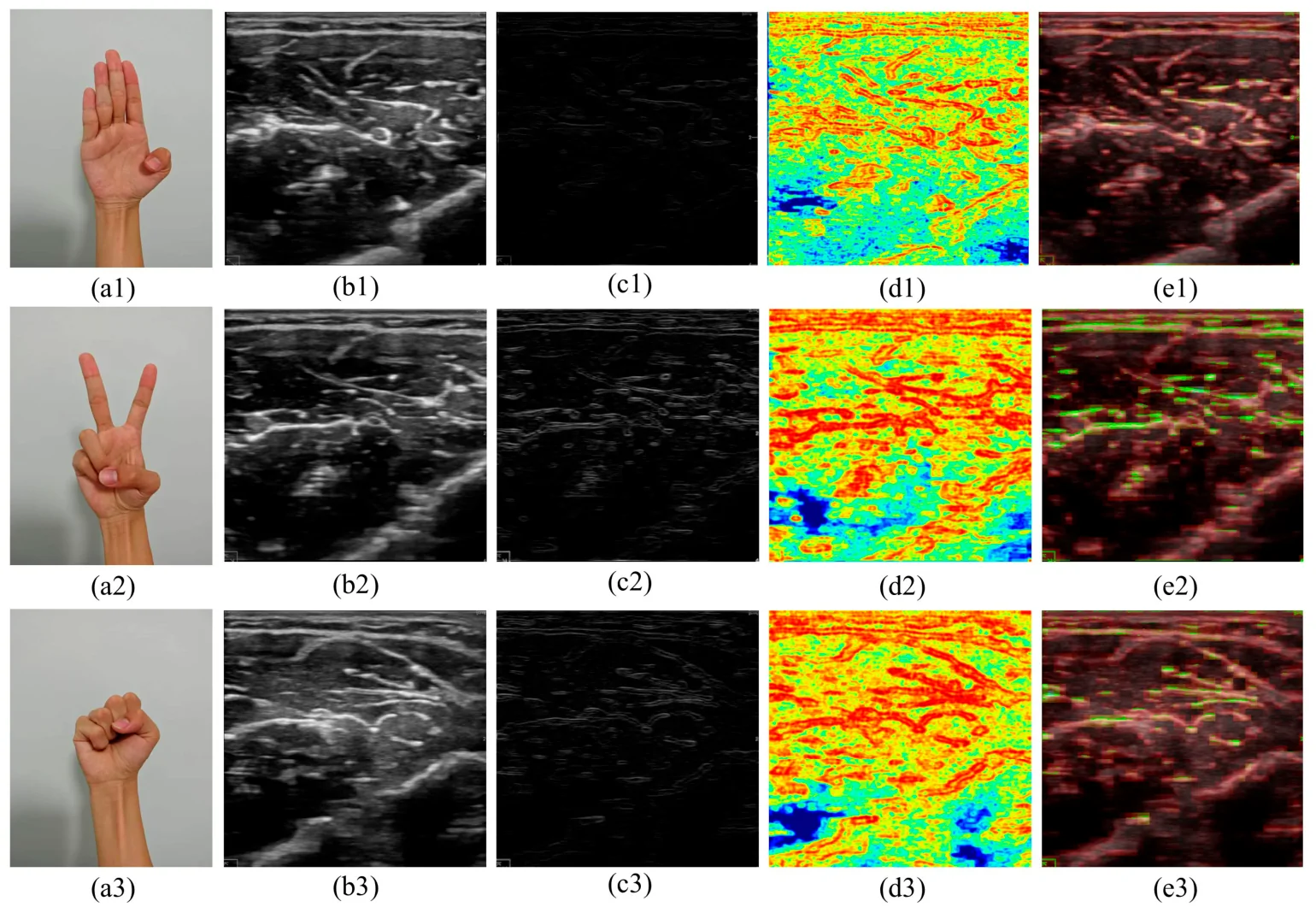
Visualization results of feature extraction for Gesture 01, 09, and 13 from Subject 2. (**a**) Gesture images; (**b**) Raw ultrasound frames of the gesture; (**c**) Gradient maps of the gesture; (**d**) Entropy heatmaps of gesture; (**e**) Feature fusion maps of the gesture.

**Figure 8:**
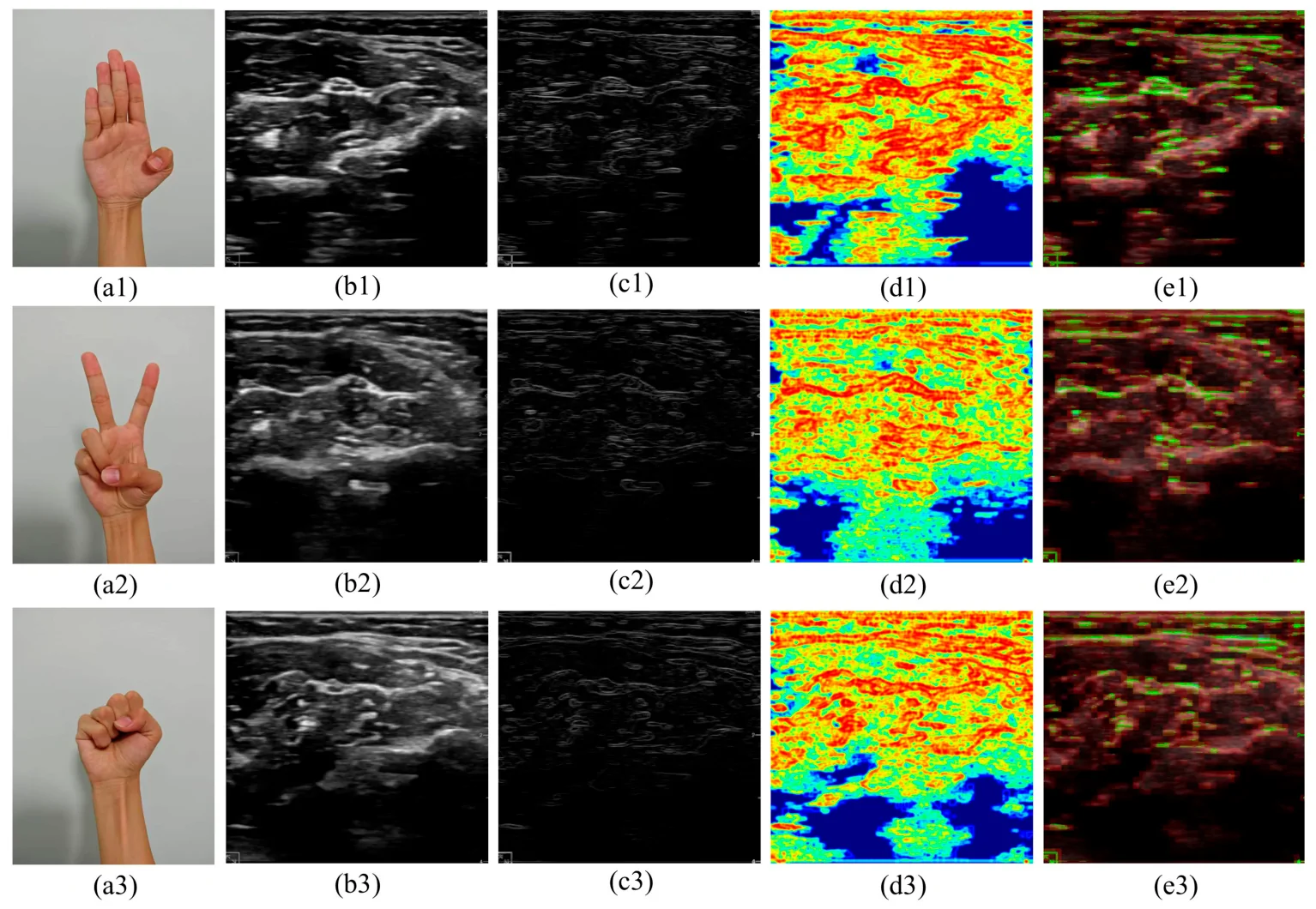
Visualization results of feature extraction for Gesture 01, 09, and 13 from Subject 3. (**a**) Gesture images; (**b**) Raw ultrasound frames of the gesture; (**c**) Gradient maps of the gesture; (**d**) Entropy heatmaps of gesture; (**e**) Feature fusion maps of the gesture.

To intuitively demonstrate the differences in muscle activation patterns across different hand gestures, Figure 6, Figure 7 and Figure 8 present the visualization results for Gesture 01, Gesture 09, and Gesture 13 from Subject 1, Subject 2 and Subject 3 respectively. For each figure, unified subplot labeling rules are adopted. Letters a, b, c, d, and e correspond to a hand gesture photo, original ultrasound frame, gradient map, entropy heatmap and fused feature map. Numerical labels 1, 2, and 3 represent Gesture 01, Gesture 09 and Gesture 13, so rows (a1)–(e1), (a2)–(e2), and (a3)–(e3) contain full feature outputs of the three target gestures in sequence.

The original ultrasound images shown in column b of each row provide the baseline muscle structure and tissue distribution. Based on these images, gradient features are extracted using the central difference method to characterize local intensity variations and structural boundaries within the muscle regions. The resulting gradient maps, shown in column c of each row, mainly highlight muscle fiber boundaries and fascia structures, which become more evident during muscle contraction. Different gestures exhibit noticeably different edge distributions and deformation patterns, indicating distinct muscle activation structures associated with specific finger motions.

Entropy heatmaps are displayed in column d of each row. Local entropy values are calculated pixel by pixel within fixed sliding neighborhood windows on ultrasound frames. The index quantifies the spatial irregularity and complexity of muscle texture. In these heatmaps, warmer colors correspond to regions with higher texture complexity and stronger local muscle activity. Cooler colors indicate relatively uniform tissue regions with minor pixel intensity fluctuation. Different gestures present distinct entropy distributions, suggesting varying levels of muscle involvement during fine hand motions.

To further illustrate the relationship among different feature representations, fused feature maps are generated by integrating gradient responses, entropy distributions, and statistical texture information with the original ultrasound images, as shown in column e of each row. The statistical features used for classification are represented by the global mean grayscale intensity and standard deviation values. Additional local standard deviation maps are constructed here only for visualization purposes to preserve spatial information. Local standard deviation values are calculated within non-overlapping image regions and visualized as statistical heatmaps. Warmer colors indicate stronger local grayscale variations and tissue reflectivity changes caused by muscle deformation. Entropy distributions further reflect local texture complexity. Green edges extracted using the central difference method emphasize the structural boundaries and deformation patterns of muscle fibers. The fused feature maps simultaneously present structural characteristics, texture complexity, and local grayscale variation patterns during different finger motions.

Consistent spatial variation rules can be observed from the visualization results of all three subjects. Different gestures exhibit distinguishable spatial patterns across gradient structures, entropy-based texture distributions, and statistical grayscale variations. These cross-subject observations demonstrate the effectiveness of the proposed multi-feature fusion strategy in characterizing gesture-specific muscle deformation information.

### 3.3. Real-Time Performance Evaluation

To evaluate the computational efficiency of the proposed recognition method, the processing time of each algorithmic stage was measured using the test dataset. As summarized in Table 2, the average feature extraction time, including image preprocessing, was 0.685 ms, whereas the RF classification required 4.478 ms. The resulting average computational latency was 5.163 ms per frame, with a maximum latency of 9.320 ms. Based on the average processing time, the corresponding computational throughput was 193.69 frames/s. This latency characterizes the computational cost of the proposed recognition algorithm, including image preprocessing, feature extraction, and RF classification, and should not be interpreted as the end-to-end response time of the complete exoskeleton system. The ultrasound imaging system operates at 30 frames/s, corresponding to an acquisition interval of approximately 33.3 ms. Thus, the average algorithmic latency of 5.163 ms is substantially shorter than the image acquisition interval, indicating that the proposed algorithm can process each acquired frame within the available acquisition period. The end-to-end system response additionally depends on the latencies associated with ultrasound image acquisition, data transmission, controller communication, and actuator dynamics. These components were not included in the present latency measurement. Therefore, the reported 5.163 ms should be regarded as the computational latency of the recognition algorithm rather than the overall response latency of the complete rehabilitation system.

### 3.4. Gesture Recognition Performance

**Figure 9:**
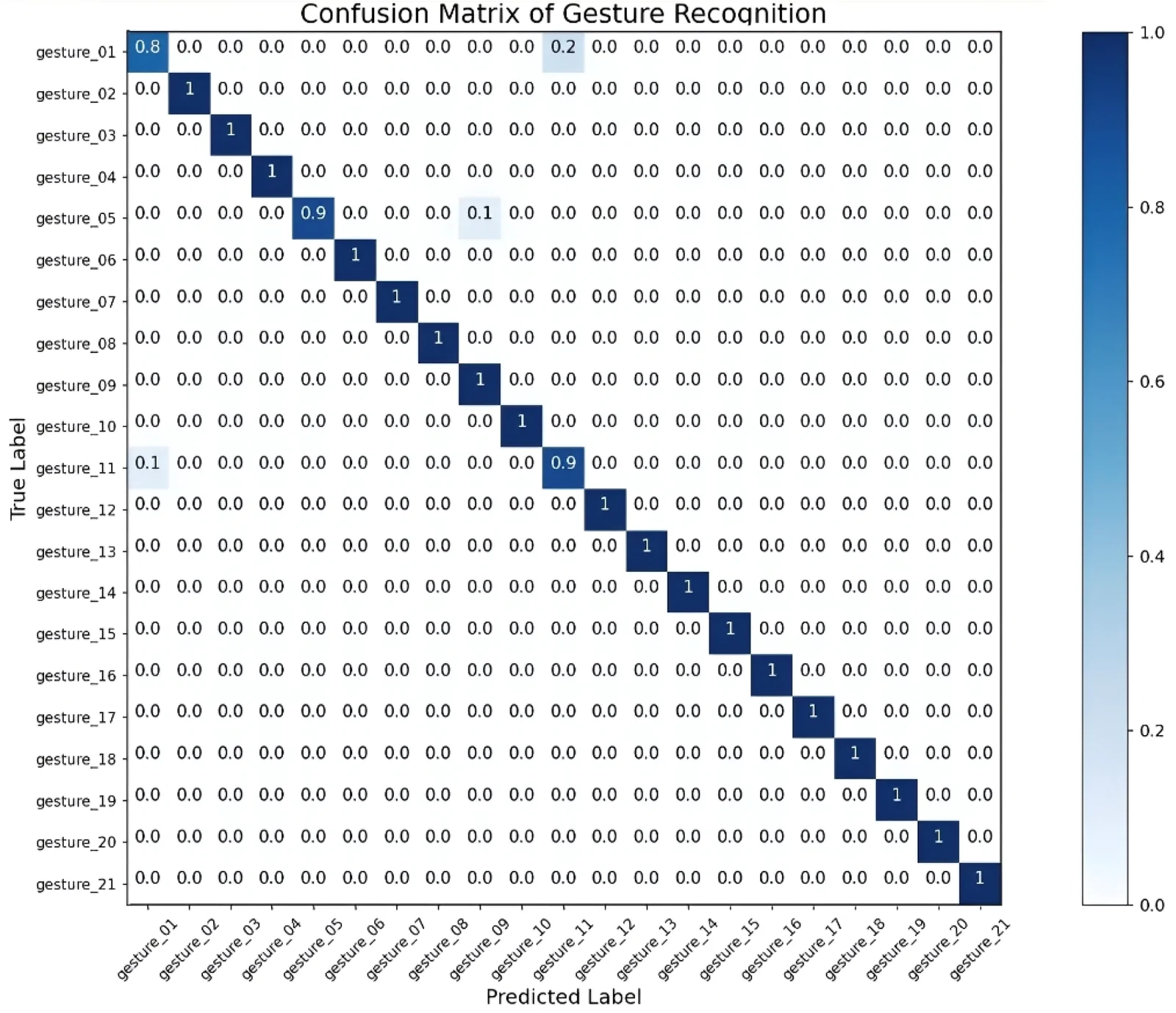
Confusion matrix of the proposed method for 21 gesture classes.

In this study, the dataset is divided into training, validation, and test sets with a ratio of 8:1:1, and the RF classifier is used to recognize the 21 kinds of hand gestures. Figure 9 illustrates the confusion matrix obtained on the testing dataset for the proposed gesture recognition system. The rows represent the ground truth labels, while the columns indicate the predicted gesture classes generated by the classifier. As shown in the figure, most samples are correctly classified, resulting in strong diagonal dominance in the confusion matrix. The majority of gesture classes achieve perfect recognition accuracy with all testing samples correctly identified. Only a few misclassification cases are observed. Specifically, 20% of the samples belonging to Gesture 01 are misclassified as Gesture 10, 10% of Gesture 05 samples are predicted as Gesture 09, and 10% of Gesture 11 samples are incorrectly classified as Gesture 01. These errors may arise from the similarity in ultrasound muscle deformation patterns between certain gestures. Overall, the confusion matrix confirms that the proposed multi-feature representation combined with the RF classifier provides reliable discrimination among different hand gesture categories.

### 3.5. Ablation Study

We conducted the ablation experiment using data from Subject 1. The experimental results further reveal the complementary characteristics among different feature types in ultrasound-based gesture recognition, as shown in Table 3. It can be observed that entropy features alone yield the lowest classification accuracy (50.00%), indicating that texture complexity by itself is insufficient to robustly distinguish different muscle deformation patterns. In contrast, gradient features significantly improve performance (80.95%), as they effectively capture structural boundaries and local spatial variations in the ultrasound images. Statistical intensity features achieve the highest accuracy among single-feature representations (93.81%), suggesting that global gray-level distribution contains more stable and discriminative information for gesture classification. When combining different feature types, consistent performance improvements are observed, confirming the complementary nature of these descriptors. In particular, combining statistical and entropy features achieves a notable accuracy of 98.00%, indicating that global intensity distribution and local texture complexity provide mutually reinforcing information. Similarly, the combination of gradient and statistical features (97.62%) also yields strong performance, suggesting that structural and intensity-based information are highly compatible for representing muscle activity patterns. However, the gain from adding entropy features to gradient-based combinations is relatively limited (94.76%), implying partial redundancy between texture and structural descriptors in this context. Finally, the fused feature representation achieves the highest accuracy (98.10%), although the improvement over the best dual-feature combination is marginal. This indicates that the proposed fusion strategy effectively integrates complementary information while approaching the upper performance bound of the current feature space. Overall, these results demonstrate that multi-domain feature integration is essential for robust ultrasound gesture recognition, while also highlighting that different feature types contribute unequally in terms of discriminative power and redundancy.

### 3.6. Comparison with Traditional Classifiers

To compare the recognition performance of different classification methods under a consistent subject-specific evaluation protocol, we conducted a benchmark comparison using three representative classifiers, including the proposed Random Forest (RF), a Support Vector Machine (SVM), and a Convolutional Neural Network (CNN) based on ResNet-18. The same subject-specific trial-level data partitioning strategy was applied to all methods to ensure a fair comparison. For image-based processing, each raw ultrasound frame was first subjected to median filtering to suppress speckle noise and then resized to a uniform 128 × 128 pixel resolution. The same preprocessing procedure was applied to the ultrasound images used for feature extraction and CNN-based recognition.

The baseline models were configured as follows. For the deep learning benchmark, a standard ResNet-18 architecture was modified for single-channel grayscale ultrasound classification by adjusting the initial convolutional layer to accept a one-channel input while retaining 64 output channels. The backbone maintained its standard four-stage residual structure with sequential channel dimensions of 64, 128, 256, and 512. Following spatial feature aggregation via global average pooling, the original fully connected layer was adapted to output 21 gesture categories. This network was trained end-to-end using the Adam optimizer and cross-entropy loss. And the SVM utilized a Radial Basis Function (RBF) kernel with a penalty parameter C=1.0. Crucially, while the CNN was trained on the preprocessed raw pixels to evaluate its autonomous feature learning capability, the SVM operated on the same multi-dimensional fused features as the proposed RF method.

**Figure 10:**
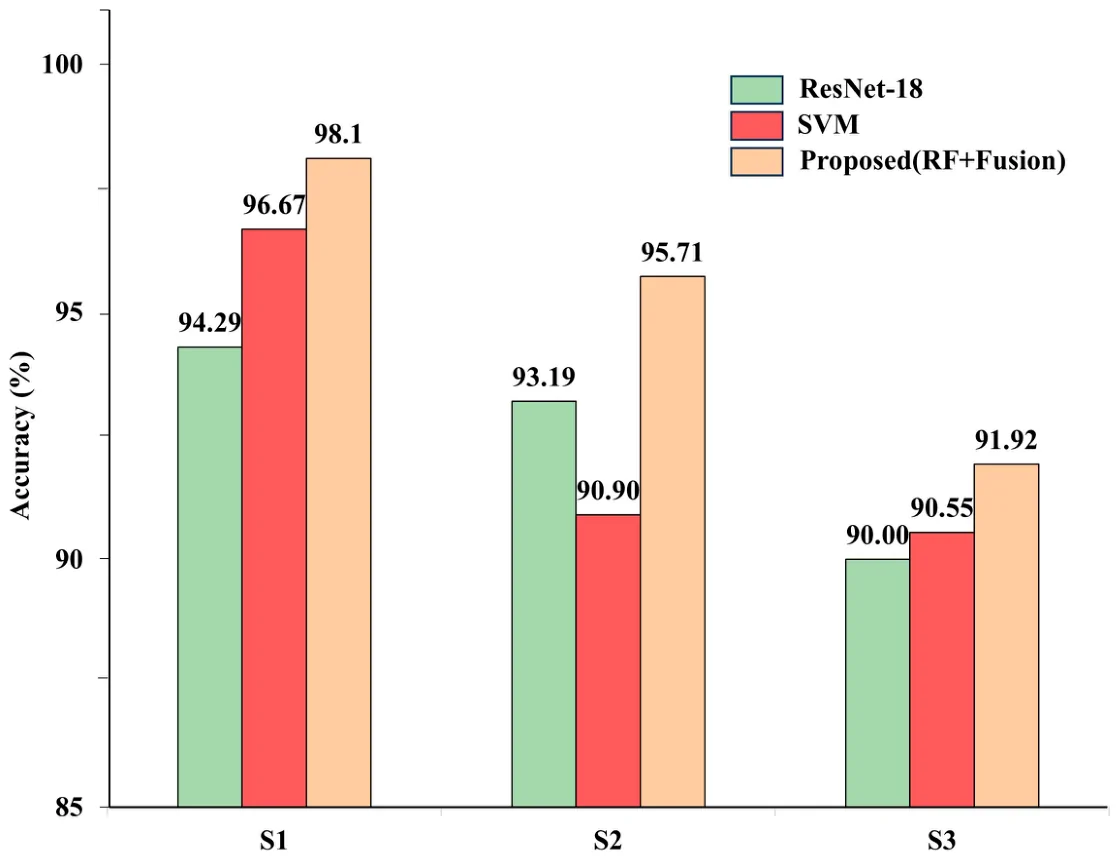
Accuracy comparison of different classification methods.

As illustrated by the accuracy metrics in Figure 10, this work compares ResNet-18, SVM and the proposed RF model. Tests cover three subjects. These participants carry clear differences in age, gender and forearm anatomical structure. ResNet-18 fails to match the performance of the two feature-driven algorithms on every tested person. This gap reveals a clear weakness of CNNs. They cannot directly extract biological muscle features from ultrasound frames. CNNs only learn spatial patterns from raw pixel values. They fail to capture physiological muscle traits. Examples include muscle fiber texture and tissue morphological gradients. Such defects become more obvious with limited training samples and variable individual forearm structures [35].

The SVM built on fused handcrafted features performs better than ResNet-18 for Subject 1, whereas its accuracy decreases substantially for Subjects 2 and 3. In comparison, the proposed RF method achieves consistently high recognition performance across all three subjects, reaching a peak accuracy of 98.10% for Subject 1. Under the subject-specific evaluation protocol, the proposed RF method achieves an average accuracy of 95.24% across the three subjects, compared with 92.71% for SVM and 92.49% for ResNet-18. These results demonstrate that the RF classifier can effectively exploit the compact, biologically relevant ultrasound features extracted in this study. Importantly, the advantage of RF in the present framework does not imply that it is intrinsically superior to deep-learning-based approaches, such as CNNs, Vision Transformers, or self-supervised learning methods. Rather, its suitability arises from the characteristics of the proposed task, as the use of low-dimensional handcrafted features reduces the dependence on large-scale training data while RF provides efficient inference with relatively low computational complexity. Combined with the low computational latency demonstrated in Section 3.3, these results indicate that the proposed feature-based RF framework provides a lightweight and computationally efficient solution for real-time ultrasound gesture recognition and is therefore well suited to the deployment requirements of soft hand exoskeleton control.

## 4. Discussion

The experimental results demonstrate that ultrasound imaging provides an effective and reliable modality for hand gesture recognition. By capturing internal muscle deformation patterns, ultrasound offers richer physiological information than surface-level signals, enabling accurate discrimination of fine finger movements. This capability is particularly important for applications requiring precise motion intention decoding.

The results indicate that different feature types contribute unequally to recognition performance. Statistical features provide the most stable and discriminative information, while gradient features capture structural variations associated with muscle contraction. Entropy features alone are less effective but offer complementary information when combined with other descriptors. These findings suggest that ultrasound images inherently contain multiple forms of informative patterns related to muscle activity. Despite the overall high recognition accuracy, the confusion matrix revealed a 20% misclassification rate between Gesture 01 and Gesture 11. A possible explanation is that these two gestures share similar activation patterns of the forearm flexor muscles. Their corresponding ultrasound images therefore exhibit partially overlapping morphological characteristics, making the subtle differences between the two gestures difficult to fully capture using the proposed fused feature representation. Although this issue has only a limited impact on the final classification accuracy, it suggests that distinguishing highly similar hand gestures remains challenging. Further improvements may be achieved by investigating more discriminative feature representations, incorporating temporal information from consecutive ultrasound frames, and expanding the training dataset.

Compared with traditional sEMG-based approaches, the proposed method benefits from the direct visualization of deep muscle structures, which reduces the influence of signal interference such as electrode displacement and crosstalk. This enables more reliable recognition of complex and fine-grained gestures. From an application perspective, the proposed approach provides a promising solution for intuitive control of hand rehabilitation exoskeletons. Accurate decoding of muscle activity from ultrasound images has the potential to support more natural human–robot interaction and facilitate future rehabilitation applications.

However, several limitations should be acknowledged. All experiments were conducted under controlled laboratory conditions, with participants performing predefined hand gestures while keeping the forearm in a relatively stable position. This protocol was adopted to isolate the relationship between muscle deformation patterns and hand-motion intentions and to facilitate stable ultrasound acquisition. Although such a constrained setup is relevant to rehabilitation scenarios in which repetitive hand exercises may be performed with the forearm supported, the effects of simultaneous forearm, wrist, or other upper-limb movements on ultrasound image characteristics and recognition performance were not systematically evaluated in the present study. Such movements may alter the relative position and orientation between the ultrasound probe and the underlying muscle tissue, change probe–skin contact conditions, or introduce additional muscle deformation patterns, thereby potentially affecting recognition performance. Therefore, the current results primarily demonstrate the feasibility of the proposed framework under controlled conditions, while further experiments under more natural and unconstrained conditions are needed to assess its robustness in practical rehabilitation scenarios.

Another limitation is that the current study included only three healthy participants. This relatively small sample size limits the representativeness of the experimental results and prevents the generalization capability of the proposed method from being fully assessed across a broader population. In addition, the muscle conditions of stroke patients may differ from those of healthy individuals. These differences may affect muscle morphology and ultrasound image characteristics, which may further influence gesture recognition performance. Therefore, the recognition accuracy obtained from the healthy participants cannot be assumed to directly represent the performance in stroke patients. Accordingly, the present study should be regarded as a preliminary proof-of-concept investigation conducted under controlled conditions, rather than a clinical validation in a stroke population. Further studies involving a larger and more diverse participant population, particularly stroke patients, are needed to systematically evaluate the robustness and clinical applicability of the proposed framework.

The proposed recognition algorithm demonstrated low computational latency in the present experiments. However, the response time of a complete rehabilitation system is also affected by ultrasound image acquisition, hardware communication, and actuator response, which were beyond the scope of this study. In addition, the current evaluation was conducted separately for each participant using a subject-specific, trial-level data partitioning strategy. Therefore, the reported average accuracy of 95.24% represents within-subject recognition performance and should not be interpreted as direct evidence of cross-subject generalization to previously unseen users. Differences in forearm anatomy, muscle morphology, and ultrasound imaging characteristics may affect recognition performance across users. For the intended rehabilitation scenario, a patient-specific model can be established or calibrated using ultrasound data collected from the target user before rehabilitation training and subsequently operated under relatively controlled conditions. Nevertheless, cross-subject generalization has not been systematically evaluated in the present study and remains an important limitation. Future work will recruit a larger and more diverse participant population and investigate subject-independent validation and adaptation strategies to further evaluate the robustness and adaptability of the proposed framework across users. Furthermore, the recognition method will be integrated into a complete rehabilitation system for end-to-end evaluation in practical rehabilitation scenarios.

## 5. Conclusions

Fine hand movement impairment frequently occurs after stroke. Existing soft hand exoskeleton control tools depend on sEMG, EEG or voice signals. These sensing approaches have obvious limits. Cross-talk, weak signal quality and environmental noise block accurate single-finger control. Ultrasound captures deep forearm muscle shape changes. It solves defects of superficial physiological sensing. Two clear gaps remain in existing ultrasound decoding research. Few algorithms link extracted features to real muscle physiological traits. Standardized ultrasound data for fine finger motion testing is scarce. This paper designs a bio-inspired ultrasound perception framework to fill these research gaps.

The first core output of this work lies in a dedicated ultrasound image dataset built for rehabilitation human–machine interaction. Twenty-one daily fine hand gestures are included. Each subject contributes 2100 standardized ultrasound frames. The data split follows an 8:1:1 trial-level rule. The split design eliminates data leakage. This dataset supplies standardized dedicated resources for follow-up fine gesture research.

A multi-scale ultrasound feature fusion scheme forms the second major contribution. Gradient descriptors, entropy texture and grayscale statistical features are extracted at the same time. These features reflect muscle fiber deformation, texture complexity and tissue reflection changes during contraction. Ablation tests confirm the three feature groups offer complementary distinguishing information. All fused features feed into an optimized Random Forest classifier. The model reaches 98.10% accuracy on Subject 1. The proposed RF method scores 95.24%, SVM averages 92.71%, and ResNet-18 averages 92.49%. Our method delivers better overall performance than the two baseline algorithms. The algorithm only takes 5.163 ms to process a single frame. This speed fully satisfies the real-time decoding requirement of the 30 Hz ultrasound acquisition system.

The whole perception framework gains full practical validation as the third key achievement. Finite state machine logic is added to the exoskeleton control loop. It filters fleeting misclassification outputs and unrealistic gesture shifts. Sudden unintended actuator movement is avoided during human–machine rehabilitation training. We carry out feature visualization, ablation comparison, cross-classifier benchmarking and latency measurement. Test results show ultrasound muscle sensing eases many drawbacks brought by traditional surface signal collection. It supports relatively stable separate decoding for single-finger delicate movements. The proposed scheme provides an alternative lightweight interaction solution for soft hand exoskeleton rehabilitation targeting post-stroke patients.

## Figures and Tables

**Table 1:** Subject information.

Subject	Age	Sex	Dominant	Medical Conditions
S1	18	Male	Right	Healthy
S2	23	Male	Right	Healthy
S3	19	Female	Right	Healthy

**Table 2:** Real-time performance evaluation of the proposed ultrasound-based hand motion recognition framework.

Metric	Value
Ultrasound acquisition rate	30 FPS
Feature extraction time	0.685 ms
RF classification latency	4.478 ms
Average computational latency	5.163 ms
Maximum computational latency	9.320 ms
Estimated computational throughput	193.69 FPS

**Table 3:** Ablation study results comparing the classification accuracy (%) of different feature types.

Feature Type	Classification Accuracy (%)
Entropy	50.00
Gradient	80.95
Statistical	93.81
Gradient + Entropy	94.76
Gradient + Statistical	97.62
Entropy + Statistical	98.00
Fused	98.10

## Data Availability

Data are available from the corresponding author upon reasonable request.

## Supplementary Materials

The following supporting information can be downloaded at: https://www.mdpi.com/article/10.3390/biomimetics11090658/s1.

## References

[1] Kuriakose D., Xiao Z. (2020). Pathophysiology and Treatment of Stroke: Present Status and Future Perspectives. Int. J. Mol. Sci..

[2] Feigin V.L., Stark B.A., Johnson C.O., Roth G.A., Bisignano C., Abady G.G., Abbasifard M., Abbasi-Kangevari M., Abd-Allah F., Abedi V. (2021). Global, Regional, and National Burden of Stroke and Its Risk Factors, 1990–2019: A Systematic Analysis for the Global Burden of Disease Study 2019. Lancet Neurol..

[3] Hmaied Assadi S., Barel H., Dudkiewicz I., Gross-Nevo R.F., Rand D. (2022). Less-Affected Hand Function Is Associated With Independence in Daily Living: A Longitudinal Study Poststroke. Stroke.

[4] Liu S., Zhang X., Zhou L., Zhang J., Liu J., He C. (2026). Robot-Assisted Therapy for Upper Limb Rehabilitation After Stroke: Umbrella Review. J. Med. Internet Res..

[5] Jin C., Chen Y., Ma Y. (2025). Effectiveness of Robot-Assisted Task-Oriented Training Intervention for Upper Limb and Daily Living Skills in Stroke Patients: A Meta-Analysis. PLoS ONE.

[6] Meng H., Zhao Z., Li S., Wang S., Wang J., Yang C., Tang C., Chen X., Zhai X., Pan Y. (2026). Active Rehabilitation Technologies for Post-Stroke Patients. Biosensors.

[7] Kang B.B., Choi H., Lee H., Cho K.-J. (2019). Exo-Glove Poly II: A Polymer-Based Soft Wearable Robot for the Hand with a Tendon-Driven Actuation System. Soft Robot..

[8] Yap H.K., Khin P.M., Koh T.H., Sun Y., Liang X., Lim J.H., Yeow C.-H. (2017). A Fully Fabric-Based Bidirectional Soft Robotic Glove for Assistance and Rehabilitation of Hand Impaired Patients. IEEE Robot. Autom. Lett..

[9] Kim B., Choi H., Kim K., Jeong S., Cho K.-J. (2025). Exo-Glove Shell: A Hybrid Rigid-Soft Wearable Robot for Thumb Opposition with an Under-Actuated Tendon-Driven System. Soft Robot..

[10] Chu C.-Y., Patterson R.M. (2018). Soft Robotic Devices for Hand Rehabilitation and Assistance: A Narrative Review. J. Neuroeng. Rehabil..

[11] Takahashi N., Takahashi H., Koike H. Soft Exoskeleton Glove Enabling Force Feedback for Human-Like Finger Posture Control with 20 Degrees of Freedom. Proceedings of the 2019 IEEE World Haptics Conference (WHC).

[12] Polygerinos P., Wang Z., Galloway K.C., Wood R.J., Walsh C.J. (2015). Soft Robotic Glove for Combined Assistance and At-Home Rehabilitation. Robot. Auton. Syst..

[13] Fischer H.C., Triandafilou K.M., Thielbar K.O., Ochoa J.M., Lazzaro E.D.C., Pacholski K.A., Kamper D.G. (2016). Use of a Portable Assistive Glove to Facilitate Rehabilitation in Stroke Survivors With Severe Hand Impairment. IEEE Trans. Neural Syst. Rehabil. Eng..

[14] Toro-Ossaba A., Tejada J.C., Sanin-Villa D. (2025). Myoelectric Control in Rehabilitative and Assistive Soft Exoskeletons: A Comprehensive Review of Trends, Challenges, and Integration with Soft Robotic Devices. Biomimetics.

[15] Gopinath D.E., Argall B.D. (2020). Active Intent Disambiguation for Shared Control Robots. IEEE Trans. Neural Syst. Rehabil. Eng..

[16] Xu H., Xiong A. (2021). Advances and Disturbances in sEMG-Based Intentions and Movements Recognition: A Review. IEEE Sens. J..

[17] Wei Z., Zhang Z.-Q., Xie S.Q. (2024). Continuous Motion Intention Prediction Using sEMG for Upper-Limb Rehabilitation: A Systematic Review of Model-Based and Model-Free Approaches. IEEE Trans. Neural Syst. Rehabil. Eng..

[18] Copaci D., Cerro D.S.D., Guadalupe J.A., Lorente L.M., Rojas D.B. (2024). sEMG-Controlled Soft Exo-Glove for Assistive Rehabilitation Therapies. IEEE Access.

[19] Secciani N., Topini A., Ridolfi A., Meli E., Allotta B. (2020). A Novel Point-in-Polygon-Based sEMG Classifier for Hand Exoskeleton Systems. IEEE Trans. Neural Syst. Rehabil. Eng..

[20] Ding Y., Udompanyawit C., Zhang Y., He B. (2025). EEG-Based Brain-Computer Interface Enables Real-Time Robotic Hand Control at Individual Finger Level. Nat. Commun..

[21] Bhagat N.A., Venkatakrishnan A., Abibullaev B., Artz E.J., Yozbatiran N., Blank A.A., French J., Karmonik C., Grossman R.G., O’Malley M.K. (2016). Design and Optimization of an EEG-Based Brain Machine Interface (BMI) to an Upper-Limb Exoskeleton for Stroke Survivors. Front. Neurosci..

[22] Randazzo L., Iturrate I., Perdikis S., Millán J.d.R. (2018). Mano: A Wearable Hand Exoskeleton for Activities of Daily Living and Neurorehabilitation. IEEE Robot. Autom. Lett..

[23] Pulikottil T., Biffi E., Diella E., Dangelo M.G., Caimmi M. (2026). Testing the Usability of a Voice Control System for Assistive Robotic Arms in People with Neurological Conditions. J. Neuroeng. Rehabil..

[24] Guo Y., Xu W., Bravo C., Ben-Tzvi P. Voice-Controlled Human-Machine Interface for an Assistive Exoskeleton Glove Aiding Patients with Brachial Plexus Injuries. Proceedings of the 2024 33rd IEEE International Conference on Robot and Human Interactive Communication (ROMAN).

[25] Tran P., Jeong S., Wolf S.L., Desai J.P. (2020). Patient-Specific, Voice-Controlled, Robotic FLEXotendon Glove-II System for Spinal Cord Injury. IEEE Robot. Autom. Lett..

[26] Chen W., Li G., Li N., Wang W., Yu P., Wang R., Xue X., Zhao X., Liu L. (2023). Restoring Voluntary Bimanual Activities of Patients With Chronic Hemiparesis Through a Foot-Controlled Hand/Forearm Exoskeleton. IEEE Trans. Neural Syst. Rehabil. Eng..

[27] Nazari F., Mohajer N., Nahavandi D., Khosravi A., Nahavandi S. (2023). Applied Exoskeleton Technology: A Comprehensive Review of Physical and Cognitive Human–Robot Interaction. IEEE Trans. Cogn. Dev. Syst..

[28] Yin Z., Chen H., Yang X., Liu Y., Zhang N., Meng J., Liu H. (2022). A Wearable Ultrasound Interface for Prosthetic Hand Control. IEEE J. Biomed. Health Inform..

[29] Li J., Zhu K., Pan L. (2022). Wrist and Finger Motion Recognition via M-Mode Ultrasound Signal: A Feasibility Study. Biomed. Signal Process. Control.

[30] Lu Z., Cai S., Chen B., Liu Z., Guo L., Yao L. (2022). Wearable Real-Time Gesture Recognition Scheme Based on A-Mode Ultrasound. IEEE Trans. Neural Syst. Rehabil. Eng..

[31] He K. (2025). Ultrasound-Based Human Machine Interfaces for Hand Gesture Recognition: A Scoping Review and Future Direction. IEEE Trans. Med. Robot. Bionics.

[32] Akhlaghi N., Baker C.A., Lahlou M., Zafar H., Murthy K.G., Rangwala H.S., Kosecka J., Joiner W.M., Pancrazio J.J., Sikdar S. (2016). Real-Time Classification of Hand Motions Using Ultrasound Imaging of Forearm Muscles. IEEE Trans. Biomed. Eng..

[33] Diogo R., Richmond B.G., Wood B. (2012). Evolution and Homologies of Primate and Modern Human Hand and Forearm Muscles, with Notes on Thumb Movements and Tool Use. J. Hum. Evol..

[34] Chen W., Li G., Li N. (2022). Soft exoskeleton with fully actuated thumb movements for grasping assistance. IEEE Trans. Robot..

[35] Vranay D., Katona M., Sinčák P. (2023). Comparative Analysis of Convolutional and Capsule Networks on Decreasing Dataset Sizes: Insights for Real-World Applications. Proceedings of the 2023 World Symposium on Digital Intelligence for Systems and Machines (DISA), Košice, Slovakia, 21–22 September 2023.

